# Fellow-Workers and Their Doings

**Published:** 1914-06-13

**Authors:** 


### ROUND THE WORLD.

### Fellow-Workers and their Doings.

**[The Editor Invites Early Information and Contributions Marked "Fellow-Workers."]**

SIR BERTRAND DAWSON, M.D., F.R.C.P., who has just been appointed physician in ordinary to H.M. the King, is not only popular among the members of the staff at the London Hospital, but also among the consultants in the West End. He was appointed to the staff of the "London" as assistant physician in 1896, and promoted to be full physician ten years later. Sir Bertrand Dawson's writings have been chiefly associated with internal medicine, and latterly he has given a good deal of attention to gastro-intestinal ailments. He was appointed physician extraordinary to H.M. the King in 1907 and created a K.C.V.O. in 1911.

DR. FREDERICK STANLEY HEWETT, who in future will share with Sir Bertrand Dawson the work formerly done by the late Sir Francis Laking, is to be congratulated on having obtained the important appointments of surgeon apothecary to their Majesties the King and Queen Alexandra. Educated at Cambridge and St. Thomas's, Dr. Hewett qualified M.R.C.S., L.R.C.P. in 1905 and became an M.D. Cantab. in 1910. Having held resident appointments at St. Thomas's and the West London Hospitals, he became attached to the apothecary's department of his Majesty's household and also took up the appointment of anæsthetist to the Victoria Hospital for Children.

LIEUT.-COLONEL LEONARD ROGERS, of the Indian Medical Service, generally illuminates whatever department of tropical medicine he turns his attention to, and his previous researches have now been added to by his work on the gastro-intestinal disorders commonly met with in hot countries. In a communication to the *Lancet* (June 6, 1914) he draws attention to the advantages of treating sprue by a combination of streptococcal vaccines and hypodermic injections of emetine hydrochloride. In the two cases now recorded severe attacks of sprue were apparently cured by a course of treatment in which half-grain doses of emetine hydrochloride were given every other day to commence with, and a streptococcal vaccine injected once a week. Lieut.-Colonel Rogers, who is now professor of pathology at the Medical College, Calcutta, is an M.D. Lond. and Fellow of both the Royal Colleges. He was created C.I.E. three years ago. It will be remembered that some of his most important work has been in connection with snake poisons.

DR. C. HUBERT ROBERTS, M.D., F.R.C.P., F.R.C.S., to whom the Local Government Board has lately given an award of £250 for his services as public vaccinator to Queen Charlotte's Hospital, is physician to that institution and to the Samaritan Hospital for Women. Dr. Roberts secured many brilliant successes in his early days at St. Bartholomew's, amongst which may be mentioned the M.D. Lond. gold medal in 1895; he was in earlier years demonstrator of practical midwifery and diseases of women as well as resident obstetrical assistant there.

UNDOUBTEDLY original work carried out by resident medical officers who can spare the time for it is to be greatly encouraged, and considerable interest attaches to an investigation which has lately been undertaken by Dr. Gordon W. Spencer, house physician to the Bristol Royal Infirmary, concerning a method of treating parasyphilitic diseases by injections of salvarsanised serum. Publishing an account of this method in the *Lancet* (May 30) Dr. Spencer gives notes of a number of cases which appeared to have been greatly benefited by the treatment, for which in his opinion there is a great future. In practice an intravenous injection of neo-salvarsan is first given, and twenty-four hours afterwards blood is withdrawn from a vein; the serum collected from this is subsequently injected into the intrathecal space around the spinal cord. Dr. Spencer is a Cambridge and St. Thomas's man, and was formerly a resident at Addenbrooke's Hospital.

DR. J. H. SEQUEIRA, who has just been appointed secretary of the British section of the new International Association of Dermatology, is well known as the head of the skin department at the London Hospital. For many years Dr. Sequeira has particularly associated himself with the development of the Finsen light treatment. Dr. Sequeira had a distinguished career as a student, and was awarded double honours at the M.B. Lond. examination in 1890. He became an F.R.C.S. Eng. by examination some twenty years ago and received the distinction of being elected to the Fellowship of the Royal College of Physicians in 1905. He holds also the appointment of dermatologist to the Radium Institute, having of late years taken an interest in radium therapy.

THE new assistant surgeon to the Children's Hospital at Newcastle-on-Tyne, Mr. David Ranken, M.S. Lond., F.R.C.S. Eng., studied medicine at Durham, St. Bartholomew's Hospital, and in Vienna. In the course of his student's career he carried off more than one important scholarship, and after qualifying in 1907 became resident medical officer to the institution where he has just been appointed a member of the surgical staff. He has also done a good deal of work in the eye, ear, and nose department of the Royal Victoria Infirmary at Newcastle.

THE Cavendish lecture of the West London Medico-Chirurgical Society will be delivered on June 19 in the Kensington Town Hall by Professor F. W. Mott, F.R.S., on "The Causes of Insanity." This distinguished Charing Cross Hospital physician has achieved worldwide fame by his pathological researches upon material derived from the London County Asylums. He is without doubt the first authority on this subject in this or any other country, and the honour which now falls to him is but one of a long succession of recognitions of his scientific eminence.

